# The little things are big: evaluation of a compassionate community approach for promoting the health of vulnerable persons

**DOI:** 10.1186/s12889-021-12256-9

**Published:** 2021-12-11

**Authors:** Kathryn Pfaff, Heather Krohn, Jamie Crawley, Michelle Howard, Pooya Moradian Zadeh, Felicia Varacalli, Padma Ravi, Deborah Sattler

**Affiliations:** 1 Faculty of Nursing, University of Windsor, Windsor, Canada; 2 Department of Family Medicine, McMaster University, Hamilton, Canada; 3 School of Computer Science, University of Windsor, Windsor, Canada; 4 Windsor-Essex Compassion Care Community, Windsor, Canada

**Keywords:** Vulnerable populations, Homeless persons, Community participation, Program evaluation, Compassionate communities, Health services research, Implementation science, Qualitative research

## Abstract

**Background:**

Vulnerable persons are individuals whose life situations create or exacerbate vulnerabilities, such as low income, housing insecurity and social isolation. Vulnerable people often receive a patchwork of health and social care services that does not appropriately address their needs. The cost of health and social care services escalate when these individuals live without appropriate supports. Compassionate Communities apply a population health theory of practice wherein citizens are mobilized along with health and social care supports to holistically address the needs of persons experiencing vulnerabilities.

**Aim:**

The purpose of this study was to evaluate the implementation of a compassionate community intervention for vulnerable persons in Windsor Ontario, Canada.

**Methods:**

This applied qualitative study was informed by the Consolidated Framework for Implementation Research. We collected and analyzed focus group and interview data from 16 program stakeholders: eight program clients, three program coordinators, two case managers from the regional health authority, one administrator from a partnering community program, and two nursing student volunteers in March through June 2018. An iterative analytic process was applied to understand what aspects of the program work where and why.

**Results:**

The findings suggest that the program acts as a safety net that supports people who are falling through the cracks of the formal care system. The ‘little things’ often had the biggest impact on client well-being and care delivery. The big and little things were achieved through three key processes: taking time, advocating for services and resources, and empowering clients to set personal health goals and make authentic community connections.

**Conclusion:**

Compassionate Communities can address the holistic, personalized, and client-centred needs of people experiencing homelessness and/or low income and social isolation. Volunteers are often untapped health and social care capital that can be mobilized to promote the health of vulnerable persons*.* Student volunteers may benefit from experiencing and responding to the needs of a community’s most vulnerable members.

**Supplementary Information:**

The online version contains supplementary material available at 10.1186/s12889-021-12256-9.

## Background

Vulnerable populations experience significant barriers accessing social, economic, political, and environmental resources [1, 2]. The result is poorer health. Without resources, these persons become unable to protect or care for themselves, either permanently or temporarily, often due to physical, mental, emotional or other causes [3, 4]. While there is debate surrounding the term ‘vulnerability’, its indicators include homelessness or housing insecurity, low-income, physical or mental frailty, social isolation, and having a physical or mental disability [3, 5]. For the purpose of this study, we use these criteria as our definition of vulnerability.

Low income is the most significant predictor of experiencing vulnerability [3, 5]. In Canada, almost one-tenth of the population experiences low income [5]. Nearly one in five Canadians who rent housing spend more than 50% of their income solely on rent [6], putting them at risk of homelessness [7]. One quarter of a million Canadians experience homelessness, and every night, 35,000 people sleep in parks and on the streets [6]. These statistics do not include the hidden homeless. The hidden homeless lack permanent housing and frequently sleep in their cars or ‘couch-surf’; the latter involves relying on family, friends for providing sleeping accommodations [8, 9]. Accordingly, 2.3 million Canadians report experiencing hidden homelessness at some point in their lives [9]. Regardless of homelessness type, these people experience significant challenges in finding a job, living a healthy lifestyle, and maintaining relationships with others [10]. People who experience homelessness are at greater risk for acute and chronic illnesses [11] and the chance of living until the age of 75 is approximately 32% in males and 60% in females [12]. Sadly, they may only receive a patchwork of health and social care services that are often not well coordinated.

Eliminating health care and social service gaps and reducing barriers to accessing care is challenging at the individual, community, and population health levels. In Canada, funding is insufficient to address the housing needs of low-income citizens, and there are inadequate numbers and availability of shelter beds [13]. People experiencing low income and homelessness often feel stigma and therefore, lack trust in providers when accessing care [14]. People who experience indicators of vulnerability may not view these indicators as problematic [3] making identification, engagement, and intervention difficult.

### The compassionate community movement

Compassionate Communities (CCs) are spreading worldwide but are relatively new in Canada. The CC movement is a population-based theory of practice that calls on society to intentionally contribute to caring for its citizens [15], especially those experiencing indicators of vulnerability. In this model, citizens are purposefully mobilized as volunteers with health and social care institutions to help people in need identify their own person-centred goals for living well. People are then connected with community resources and empowered to act on their goals and needs. With collective engagement, a CC becomes an interplay of caring actions with and among a community, its citizens, and health/social care organizations [15].

In Canada, the CC movement is led by a collective of palliative care stakeholder organizations [16–18], but the approach is adaptable for people of wide-ranging health needs and vulnerabilities. The CC theory of practice can be implemented to best suit a community’s priorities, needs, and resources. When strategically put into practice, CCs can improve the quality of life for persons living with precarious health, social and environmental circumstances [19].

### The Windsor-Essex compassion care community

The Windsor-Essex Compassion Care Community (WECCC) is a collective of volunteers and 65 health/social care organizations that partner in identifying and reducing the unmet needs of persons living with complex health and social issues [19]. Target populations include seniors, the frail elderly, people with chronic disease and disabilities, and people living in social isolation. WECCC staff and volunteers assist clients to identify their own personal needs, goals, and preferred interventions.

The Vulnerable Persons (VP) Program, a sub-project of the WECCC, was born out of a need to provide focused support for people living with low income and housing insecurity in Windsor-Essex, Ontario Canada. In collaboration with the regional health authority, Family Services Windsor-Essex, the Hospice of Windsor and Essex County, the primary care sector and others, VP program staff and volunteers have worked with over 400 individuals to develop goals that address their unmet health and social needs. Clients are never discharged, and service level is determined by client need. Programming varies from face-to-face intervention with fully integrated health and social care supports, to scheduled check-in calls by staff and volunteers for assessing client goal achievement and quality of life.

Currently, little is known about the experiences of CC stakeholders and how to successfully implement CCs among vulnerable persons. This information is needed to improve and spread this program and to inform others who are implementing similar initiatives. The purpose of this exploratory study was to evaluate the implementation of a compassionate community intervention for vulnerable persons in Windsor Ontario, Canada. In particular, we sought to describe and interpret stakeholder experiences about the program’s characteristics, its processes, and potential impacts and opportunities.

## Methods

We employed an applied qualitative approach [20] to describe and interpret stakeholder perspectives about the VP program. This approach enabled us to critically examine the data to develop a rich understanding of the stakeholder experiences, the program’s processes, its impacts, and areas for improvement. WECCC’s research and evaluation program is guided by constructs of the Consolidated Framework for Implementation Research (CFIR) [21, 22]. In this evaluation we focused on several constructs within its domains - characteristics of individuals, intervention characteristics, outer setting, and program processes [21]. We deemed them to be the core domains on which to focus for understanding the ‘what’ and ‘how’ of the VP program implementation.

### Sample and recruitment

We used convenience sampling to identify individuals who met the following criteria: (1) being either a program client *or* a stakeholder who is actively engaged in program delivery, (2) over the age of 18 and (3) English speaking. Participants were recruited by WECCC office staff using a structured script over a four-month period of time between March and June 2018. The final sample included 16 program stakeholders made up of three VP coordinators, two community case managers from the regional health authority, one administrator of a key partner community program, two nursing student volunteers who had completed a community clinical experience with the VP program, and eight VP clients. Among the VP clients, one person was experiencing homelessness at the time of data collection. The remaining clients were previously homeless but living in temporary and/or precarious living situations.

### Data collection

We conducted one focus group with five VP clients and individual telephone interviews with three clients who were unable to attend the focus group. A focus group was purposefully selected as we sought to gather and validate collective client perspectives about the program. The focus group took place at the Hospice of Windsor and Essex County and transportation to the Hospice was provided for VP clients. It was facilitated by JC. Field notes were documented by HK and observations noted by KP. Individual telephone interviews were also completed with the VP care coordinators, the community care case managers, the partner program administrator, and the student volunteers. These interviews were completed by JC, HK, and KP. The interview guide was developed for this study with questions and prompts informed by the CFIR Interview Guide tool [21]. Refer to Supplementary 1. The same interview schedule was used for all stakeholders. .

All focus group and interview data were digitally audio-recorded and transcribed verbatim by a trained transcriptionist and research assistants. Two VP coordinators and three participants agreed to engage in member check interviews in which we shared the emerging themes and invited them to confirm, disconfirm, and offer further explanations. There was agreement from participants regarding the emerging findings. We confirmed data redundancy for the overall themes and therefore ceased data collection.

### Analysis

We applied Sally Thorne’s pragmatic approach to selecting data analysis procedures [20]. Three nursing researchers (KP, HK and JC) and a research assistant (FV) iteratively reviewed the transcripts individually. We met as a team to discuss early insights and potential codes. A codebook was established to support early coding and researcher consistency with coding. During open coding, new codes were created and documented on the transcripts by each member of the team. We simultaneously extracted meaningful/powerful quotes to a shared word document. During weekly team meetings, codes were reviewed, revised and some were abandoned as they were deemed to not reflect the data. Throughout the process, we applied a constant comparative approach [23] to the data comparison, and re-organization of the data into categories that were later collapsed into emerging themes. During this time, we considered a range of possibilities, took care to avoid premature closure [20] and documented our decisions. We met weekly in the last 3 weeks of analysis to agree upon the overall theme and its sub-categories. We collated and shared our individual memos and analytic insights in face-to-face discussions, and then mapped these notes to our themes as a method for validating our interpretations. Decisions were reached by consensus and the findings were unanimously approved by the team.

### Ethical considerations

Ethics clearance was granted by the University of Windsor Research Ethics Board (REB# 16–047). Consent was gathered and documented individually for all participants in both the focus groups and the interviews. All participants were invited to create and share their preferred pseudonyms and to ask any questions of the researchers before beginning the focus groups and interviews. Focus group participants were reminded that confidentiality could not be assured due to the group nature of data collection, but participants agreed to not share information provided by others. Client participants were assured that their decision to participate (or not participate) would have no impact on their program services.

## Results

The findings are organized and presented in the following sections: (1) participant characteristics, (2) intervention characteristics, (3) program processes, and (4) impacts and opportunities for improvement.

### Participant characteristics

Vulnerable clients were described (by self and providers) as “invisible” within the system and being socially isolated. They were also characterized by providers as having “brittle support systems”, being disconnected from family, and having “no one looking out for them.” Clients and providers described complex health issues that include, but were not limited to developmental disabilities, anxiety, depression, renal failure, immobility, and pain. Life challenges that prompted referral to the program included homelessness, financial insecurity, elder abuse, bereavement, and caregiver burden. As stated by one care coordinator: *“Life has kind of dealt them a crappy hand. A lot of times it’s about the social determinants of health and some people just aren’t as privileged as others … and there’s just not the supports in place, or there are supports but they’re not readily acceptable to people and it prevents them from really getting the help that they need …*” (VP Coordinator 2).

*Intervention Characteristics - The Little Things are Big.*

The analysis revealed one overarching meta-theme that describes the characteristics of the intervention, *‘the little things are big’*. Clients and providers frequently referred to the program’s ability to address ‘*the little things*’ that often go unnoticed at the systems level, but that have a big impact on client health and quality of life: *“We have fairly large caseloads and we don’t have like the days to spend working on the smaller tasks that are big for our patient. Like we put in the care plans, we put in the services for them but...it was the little things, like she [the client] wasn’t able to wash her hair and her daughter was burning out and didn’t have contact with anyone in the community.”* (Community case manager).

The ‘*little things’* commonly involved assisting with personal and practical needs (personal care, groceries, meal preparation, finances, home maintenance) that kept clients healthy, safe, and in some cases prevented them from being evicted and/or being able to remain in their homes. *“I have a gentleman that I’m working with, he’s got ALS [Amyotrophic Lateral Sclerosis] and he needs somebody to go to his house and just help him get his lunch from his stove to his table, that’s it. I mean it seems like such a simple thing, but he had a great deal with difficultly doing that”* (VP care coordinator 1). Jane, a VP client, shared the following: *“He helped me with my portable air conditioner, setting it up and so we got it running...I have a medical alert button and he put it all together...and made a phone call that took like an hour with these people, but he saved me $300 …”.*

Social support was perceived as a little but significant thing for clients, staff and students *“A lot of people think you have to constantly be doing physical things for people or sending referrals, but a lot of people just want someone to talk to … especially vulnerable people who don’t often get the opportunity to just sit and chat with somebody. It is very beneficial, and I love seeing people’s lives change, specifically for the better, just through the support that we’re able to give them”* (VP coordinator 2).

Some clients received friendly visits from WECCC volunteers and/or were connected with WECCC’s community partner programs. All participants described the importance of therapeutic communication and listening skills as being the core component of the program intervention. Molly stated: *“That was a comfort to me to know that there were individuals concerned with little, old me in the sense that … we all come from different parts of a community and they’ve included everybody and that’s a very emotional thing for me to have support from people that I don’t even know.”*

Clients receive ongoing, free support and this was perceived as a “big thing” for clients and the program.

*“Obviously there’s no cost so that’s a big thing, but we never tell them they’re discharged from our program so we can see them once a week until* *they* feel *supported which I think is a huge relief for them because they don’t want to tell us their whole story, be done with us in four weeks and then move on to their next worker. So that’s a big thing for us. Even when … we’re not seeing them on a weekly basis, we continue check-in calls whether that be monthly, six months, one year so it’s a huge relief for them … to know they always have our support* (VP coordinator 3).

### Program processes

The little and big things are addressed through three key processes: (1) taking time, (2) advocacy, and (3) empowerment. Each process is reported in the following text.

### Taking time

Taking time was a key process that enabled the sub-process of advocacy and empowerment: “… *when we go to a home, we’re having a conversation with the client at a pace that’s appropriate to them with intentions of building a report with client and in doing so, they begin opening up about things that they want to work on, difficulties that they’re having that they often have not shared with other people...*” (VP coordinator 1). The importance of this process was echoed by another care coordinator: *“Most other professionals that go out to people’s homes, they are so focused … that it’s a pretty quick conversation. Whereas our conversations are much more open ended … ‘so what is it that you think you could do? What would you need to improve [your] quality of life?’ This is a very big question and is not trying to fit their answer into some predetermined kind of things that you can offer. So, it takes time”* (VP coordinator, 2).

Time spent listening and communicating therapeutically was highly valued, whether it occurred face-to-face or by telephone - “*I appreciate how they don’t just [say] o.k. here is what we talked about last visit and drop a bunch of papers in front of you and you know it’s all curt like it is with a lot of offices you know. They take the time to discuss with you between your options which ones are best for you …”* (Hunch).

Receiving a monthly check-in call was the most frequently valued intervention reported by clients and providers. In some cases, the call filled a gap when other services had run out and offered a sense of security and social connectedness: *The client asked me when I talked to him last, ‘Would you be able to call and just check up to make sure I’m doing okay? Can you please call me in a month just to check in?’... So, I’ll call again in another month* (VP coordinator 2)*.*

### Advocacy

The process of advocacy encompassed activities such as researching programs and services, contacting providers and community organizations, and explaining the client’s complex health and living situation. Advocacy work was successful in securing vital care and services, such as free and/or affordable transportation for clients, funding for medical equipment, prescription medications, assistance with activities of daily living, and temporary housing.

A community case manager from the regional health authority described the following example of advocacy work:

*“I have this mid 70s lady who falls into the category of having a brittle support system, had a fire in the summer in her condo, she’s on hemodialysis. She … was missing dialysis a lot, was going to the ER with shortness of breath … A constant ride to dialysis was the reason she was missing it plus she was suffering some depression … It took a lot of coordination, but we were able to get her rides. I was able to get her providers to start early, to get her ready for dialysis, get her on and off transport … WECCC dug deeper and was able to connect with the social worker and found funding to get this ride and now her dialysis times have been changed... The patient is now going to dialysis*.”

Greg shared an example of advocacy when facing homelessness after being discharged following a recent hospitalization: *“I’ve been [living] with a broken back over 10 years ago when I went backwards down the basement stairs … I ended up with a fractured skull and a cerebral hemorrhage … He* [VP care coordinator] *helped me find a place and he booked me in a* [rest home] *for about nine months … and did some work on getting me an electric scooter …*. [*my] mobility is not getting better...”*

### Empowerment

Writing personal health goals was identified as the key process for client empowerment by six clients and all of the other stakeholders: *“I think the most important part of it is the establishing of SMART [Specific, Measurable, Attainable, Relevant, Time-Bound] goals. Those provide direction and they also help actually motivate the clients to achieve the goal that they have identified.”* (VP coordinator 1).

Some participants were affirmed by the power of goal setting for clients living in precarious life circumstances. Shawn explained: *“You would like never put that two and two together yourself but to have somebody say to you ‘Yes you know this is something that you can do.’ That just makes you feel productive as a person, definitely.”* The program administrator from a partner community program shared the following: *“One of our clients who is palliative … there’s a persistent level of depression … but you know she was still able to make some goals. She was still able identify that ‘I would want to do this, this and this’ before it all ends for her.”*

The nursing students validated the value and power of goal setting for empowerment. Brianna explained: “*You’re asking them ‘what can you do to improve your quality of life?’...and it helps people realize, ‘Oh I can change this. I don’t want this to be my life the way it is’ and we help with figuring it out … A lot of the time too we would help make goals for the patients and say, ‘o.k. we’ll do this to help you get to here’ and by the time we called the next week... they’ve done it on their own.”*

Making social connections was one of the most identified goals reported by clients and staff. *“Getting out more is the number one reason people are referred to us, it’s just people are so isolated and so getting out more is one of the biggest goals”* (VP Coordinator 2). Clients were empowered to improve their social connections and their personal well-being through intentional connections to community activities, such as card groups and yoga. *“We’re empowering them to create one linkage that leads to the next in the community so just getting them involved in other programs so they have some kind of care circle in their life”* (VP Coordinator 3). Transportation provided by the local hospice enabled attendance at some programs. *“You know I get to meet people and get out of here...with the rheumatoid I wake up with pain every day...my goal is to get back swimming … See if that benefits it [the pain]. There’s other things Hospice offers, other programs, ‘Living with Chronic Pain’ is one of them”* (Greg).

A few clients were empowered to use their talents to give back to the community. *“We had one client who was good at knitting or crocheting so we suggested that maybe she find a program where she could knit, knit hats for babies... … we did have a few clients who were heavily involved in advocacy for low income people as well as homeless people …* (nursing student volunteer). One participant described how she made woven mats from plastic milk bags for people living on the streets, and another participant set up a Facebook group to promote social connection and advocacy for the homeless and those at risk for homelessness.

### Impacts and opportunities for improvement

The qualitative data suggest positive health impacts for clients and benefits for the community and the health care system. In most situations, the program serves as a safety net that supports people who are falling through the cracks of the formal care system.

*“One of our clients, who had a stroke, she lost function in her right arm and her right leg … she was given a manual wheel chair and for two years she lived in an environment in which she was literally going in circles right because she didn’t have use in one of her arms in order to keep this wheel chair straight and she lived like that for two years! To me that sounded like an absolute system failure but one that really could have been avoided had she called the system, called the LHIN, called the doctor, any of these kinds of people who would’ve been able to intervene and should have intervened but she didn’t … if our clients are receiving monthly check-in calls, something like that will not happen*.” (VP Coordinator 1)

All of the clients reported benefits from increased social interaction and a connection with their community***.*** The participants described multiple examples of how the program is directly reducing use of emergency services, preventing homelessness, improving client safety, and in a few cases, averting attempted and completed suicides.

*“One member tried to kill himself … When he was released, he was sent to our program through the social worker. He had no other supports just himself and he lives with his brother, so we set him up with the crisis number and made a goal for him and his brother to be a support system for one another. They get out walking at least once a week and they hold each other accountable … He also wanted to work part time … so I gave him a number to the unemployment centre in his area, and he reached out to them himself … created his own resume, and he actually landed himself a job”* (VP coordinator 3).

Although long-term community investments are needed, short-term support for securing safe housing and preventing homelessness was reported as a positive impact of the program: One client with hoarding behaviours described how the program enabled her to avoid eviction by negotiating a plan to reduce the clutter: “*I had the fire marshal come in here … my house was ransacked and then I just let it go because I suffer from depression … and alcoholism. She’s (the landlord) given me like a week to get one room done and a week to get another room done and he’s [the volunteer] helping me out … He’s helped me out, period!* (Jane).

All eight clients reported support for managing chronic health issues such as pain, anxiety, depression, renal failure, and diabetes. Coordinators were able to assist clients to access dialysis appointments, prescription medications, and primary care in cases where clients had no family physician. In some situations, the coordinator attended primary care visits to add context to the situation. A nursing student volunteer discussed success with helping a client navigate management of her chronic pain:

*“I had a patient who was in chronic pain and she had no, she didn’t have a family doctor, she didn’t have any management of her pain at all. She had tried non-pharmacological things and it wasn’t working, so she was sleeping until 2:00 pm every day and then going to bed early cause … she couldn’t function … Her main thing was figuring out that pain … she was so socially isolated … because she couldn’t handle it … We got her a family doctor. We had VON* [Victorian Order of Nurses] *connect with her to help with pain management and we also signed her up with hospice … so that when she had that pain managed then we can work around the social isolation which was getting her involved with the wellness program in the community.”*

Through this evaluation, we learned from care coordinators and volunteers that training programs should include specific content and tools for responding to the needs of individuals experiencing complex mental health concerns. Sustainability opportunities include technology, funding, and volunteers. Many clients do not have internet, electronic devices, and/or are not tech savvy. Permanent funding for program coordination and volunteer training will be essential for long-term sustainability. As stated by one coordinator: *“I think the main resource that we require more than anything is volunteers … I think that is the key to it all. For myself, I am struggling to keep up just because of the kind of manpower issue. And the main reason for that … you don’t have a volunteer base big enough in order to be able to have that time.*”

## Discussion

This study is important as it adds to the growing international evidence about the positive impacts of CCs on individual and community health. The majority of the evidence is published in the palliative and end-of-life care literature [24]. This is the first study to evaluate a CC approach within a targeted vulnerable care sector. The results of this evaluation demonstrate that health and social care sectors can be mobilized by a CC approach to holistically address the big and little needs of society’s most vulnerable and invisible persons. The qualitative findings suggest that the ‘little things’ often had the biggest impact on client well-being and on care management. The ‘*big and little things’* characterize the VP intervention, and they were addressed through the processes of taking time, advocacy and empowerment. In this study, these processes appear to address vulnerabilities, such as housing security, physical and mental disabilities, and social isolation. They also meaningfully address the holistic concerns that were most pressing and important to program clients, with social isolation being a significant concern. Recruiting and retaining volunteers is the most key opportunity for improvement and sustainability of the program.

The Canadian healthcare system and those of other countries remain entrenched in approaches that are largely siloed and not coordinated to meaningfully address the things that are most valued by people and that contribute to their quality of life [25, 26]. It is overtime for policy experts and governments to prioritize an integrated system of health and social care that takes action on the ‘little things’ that often have a big impact on health. Food, transportation, safety, and social connectedness were described by participants as “little”. Yet, they are basic human needs that are essential for health and quality of life, and they are frequently not accessible to those facing vulnerabilities [27].

In Canada, there are very few community-based programs that provide holistic, and continuous life-long support for people who experience homelessness, housing insecurity and low income, and some of these programs are often criticized for reinforcing obstacles to engagement [28]. International community-based programs face similar criticisms. Many have criteria that are based on age or gender [29, 30], chronic and advanced disease [31–33], and/or on addiction or mental health issues [31, 33–35]. Others focus on interactive educational workshops and are time-limited or transitional [35]. Program benefits are often not maintained long-term [29, 32, 34] with clients reverting back to their original behaviours or circumstances after support is withdrawn. The VP program addresses these gaps and challenges by purposefully organizing communities to act on the big and little things as an issue of public health through its key processes.

### Enacting the key processes

Taking time, advocacy, and client empowerment are processes that can be readily enacted at the community level, but we argue that the processes must be systematically integrated into the system to be effective and sustainable. This systems-level change will require reconsideration of funding and service delivery, not just a shuffling of deck chairs [36] or one-off programs. CCs address these barriers by adopting a public health approach that seeks to truly understand what is most important to people where they live and by engaging people and communities to act on addressing the needs of people experiencing various health and social care issues [15, 37]. Effective, resilient, and sustainable health and social care systems can be achieved when vulnerable persons are empowered as active advisors and partners in re-shaping change [2]. The process theme of empowerment resonates with this notion in that people with vulnerabilities were empowered to act on their own goals, and support others in their own community.

Advocacy is an important public health tool for addressing the social determinants of health and an important process for addressing care gaps and inequities within the system [38]. Unfortunately, advocacy takes time, and time is a scarce resource for many health care providers [39]. Current healthcare systems reward efficiency and larger volumes of clients [40], potentially discouraging providers from taking the needed time to address a person’s holistic care needs. Mounting scholarly CC evidence is showing that caring interactions among persons, families, neighbours, and healthcare providers enable participatory care [37, 41], improve overall wellbeing and reduce mortality [37, 41, 42], and ease the burden on the care system [41]. This study supports and adds to this body of evidence.

In a CC model, volunteers are often untapped health and social care capital who have time to give back to their communities and can be mobilized in action [43]. The findings of this study again support the notion that volunteers can be equipped with the skills needed to advocate for and empower people who are experiencing vulnerabilities. At the time of this study, the VP program had 50 trained volunteers, including students from nursing, social work, and gerontology. Twenty additional volunteers were trained during the first 6 months of the COVID-19 pandemic to provide virtual check-in visits with VP clients. Because negative or inaccurate perceptions of homelessness, poverty and other types of vulnerabilities can result in deficit versus strength-based practices that further stigmatize clients [44], formal training is essential. VP volunteers are overseen by the VP care coordinators, and they participate in mandatory and specific volunteer training in areas such as diversity, communication in complex client scenarios, goal setting, the importance of socially connectedness, and self-care.

With regard to both empowerment and advocacy, this program provides a window through which nursing student volunteers viewed the realities of those experiencing vulnerabilities, and an opportunity to integrate service learning into CCs. Nursing students reported similar benefits to those described by Knecht and Fischer - shattering their own stereotypes, reciprocal learning through relational practice, and developing skills in community advocacy [45]. An added benefit of student volunteers is that they are truly able to take the time to comprehensively assess and address the breadth and depth of client needs – time that is not typically available in other clinical learning settings [45].

### Addressing social isolation

The need for improved social connectedness among VP clients is an important secondary finding of this study. Social isolation has been deemed a public health pandemic [46], a predictor of early mortality [40] and a common experience of all clients in this study. Social exclusion negatively affects the subjective wellbeing of those who experience homelessness, and interventions that engage people in building social connections will improve their quality of life [37]. Overcoming social isolation by expanding social networks is a key focus of the VP program, and its approach is informed by good evidence [40, 47, 48]. The VP program engages high risk individuals as active participants rather than passive recipients and empowers their participation in the planning and implementation of social engagement. Support is flexible and adaptable to the needs and goals of the participants, and it is rooted in both the community and the person’s own social network. An unintended and serendipitous benefit of the focus group was that participants identified creative opportunities for supporting the local homeless community and agreed to share contact information as a way of staying connected after the study concluded.

### Embracing discomfort

As stated by Karen Armstrong, founder of the global Charter for Compassion.

movement, “A compassionate city is an uncomfortable city! A city that is uncomfortable when anyone is homeless and hungry …” [49]. We add that a compassionate community should also be uncomfortable when any citizen is socially isolated or lonely. As the socioeconomic inequalities in health and other indicators of vulnerability continue to widen in Canada [50] and around the world [2, 25], it is time for every community to be very uncomfortable – uncomfortable to the point that every citizen is treated “as we would wish to be treated” [49] and empowered to take the time to take action on inequities.

### Limitations and future research

This WECCC VP program was evaluated through in-depth interview evaluations of only 16 key stakeholders. As this was an exploratory study, we did not seek to interpret variations in the experiences of clients and other stakeholders. Sampling was convenient, and only one participant was experiencing homelessness at the time of the interviews. Therefore, the findings are not transferable to those experiencing homelessness. We were unable to interview volunteers other than the nursing student volunteers. Future studies should assess and compare more stakeholder voices (community volunteers, student learners, community partners, and homeless clients) to discover other unknown benefits and additional opportunities for improvements and program scale. Because sustainability is a challenge in many community-based programs [28, 31, 33], longitudinal studies should appraise whether the impacts of the program are sustained in the long-term. A larger study using an implementation science framework would more rigorously evaluate the objective strengths and improvements of this program. Members of our research team (PMZ and KP) are applying social network analysis procedures to model WECCC as a care system. The goal is to expand the quantity and quality of social interactions in this network and others by identifying socially isolated members and those with weak social links [51]. This identification may enable communities to facilitate connections for those experiencing vulnerability and predict future social needs.

## Conclusion

This study reports stakeholder experiences of a pilot CC intervention for vulnerable persons, the program characteristics, its processes, impacts, and opportunities for improvement. Health and social care sectors can be mobilized by a CC approach to holistically address the big and little needs of society’s most vulnerable persons. These needs are physical, practical, and psychosocial. Successful implementation processes of the VP program involve taking time, empowering clients to set their own goals based on personal preference and advocating for appropriate resources that support holistic health and wellbeing. Programs for persons who are experiencing vulnerability should be client versus provider driven and provide relational support for combating social isolation. Student volunteers may benefit from learning about and responding to the needs of a community’s most vulnerable members. Sustainability opportunities include technology, funding, and volunteers. CCs are a workable solution for preventing and reducing vulnerability.

## Supplementary Information

**Additional file 1.**

## Data Availability

The data material used in this study are available from the corresponding author on reasonable request which will not conflict with the anonymity and confidentiality of the data.

## Abbreviations

CC: Compassionate Communities
VP: Vulnerable persons
WECCC: Windsor-Essex Compassion Care Community
CFIR: Consolidated Framework for Implementation Research
