# Supplementary material for: The little things are big: evaluation of a compassionate community approach for promoting the health of vulnerable persons
Source: BMC Public Health. 2021 Dec 11;21:2253. doi: 10.1186/s12889-021-12256-9 (PMC8665321; doi:10.1186/s12889-021-12256-9)
Supplement: Supplementary file 1 — Additional file 1. [file 12889_2021_12256_MOESM1_ESM.docx]

Supplementary File 1. Focus Group and Interview Guide (all stakeholders)

| Question Number | Questions and Prompts |
| --- | --- |
| 1 | Overall, what has it been like for you to be involved in the WECCC VP program? |
| 2 | In what ways has the program helped you/your clients?   - Are there any parts of the program that are most helpful, for example, coaching visits, completing a personal care plan, making connections with people, community services or other resources, and/or other ways. - Please share any specific stories about how the program has helped you/your clients? |
| 3 | How well are your/your client’s personal needs, preferences, and goals being met?   - Please share any specific examples or stories about how your/your clients’ needs, preferences and goals are or were being met by the program. |
| 4 | How does the WECCC VP program compare to other programs you have used or are using?   - What does it offer you/your client that other programs don’t or can’t? |
| 5 | Are there things that the program wasn’t or isn’t able to help you/your clients with?   - Why do you think this is the case? - Did you experience any barriers with the program? - Please share your suggestions for improving the program. |
| 6 | What do you most value about your community and do you have any talents that you could use to give back to others? |
